# Development and validation of food frequency questionnaire for food and nutrient intakes of adults in Butajira, Southern Ethiopia

**DOI:** 10.1017/jns.2021.94

**Published:** 2021-11-22

**Authors:** Ilili F. Regassa, Bilal S. Endris, Esete Habtemariam, Hamid Y. Hassen, Seifu H. Ghebreyesus

**Affiliations:** 1 Department of Nutrition and Dietetics, School of Public Health, College of Health Sciences Addis Ababa University, Addis Ababa, Ethiopia; 2 Department of Primary and interdisciplinary care, Faculty of Medicine and health sciences, University of Antwerp, 2610, Antwerp, Belgium

**Keywords:** Food frequency questionnaire, Validation, 24-HR, Adults, 24-HR, 24-h recall, FFQ, Food Frequency Questionnaire, NCD, non-communicable disease

## Abstract

We developed a culturally-specific Food Frequency Questionnaire (FFQ) to the Ethiopian context and evaluate its validity in comparison to two 24-h dietary recalls (24-HRs) of food and nutrient intake. To evaluate the validity of a culturally-specific FFQ against two 24-HRs, we used a paired *t*-test, Wilcoxon-signed-rank test, Correlation coefficients, cross-classification, *κ* and Bland-Altman analysis. The FFQ was obtained 15 d after the second 24-HR was completed. A total of 105 adults, of which 43 (41 %) were men and 62 (59 %) women, aged 20–65 years participated in this present study. Mean energy and macronutrient intake obtained from the FFQ were significantly higher than those obtained from the mean of two 24-HRs. For energy and nutrient intakes, the crude correlation ranged from 0⋅05 (total fat) to 0⋅49 (vitamin B1). The de-attenuated correlation ranged from to 0⋅10 (total fat) to 0⋅80 (vitamin A). For the majority of food groups, no significant difference was observed in the median intake of food and nutrients. Crude correlation for food groups ranged from 0⋅12 (egg) to 0⋅78 (legumes). The de-attenuated correlation ranged from 0⋅24 (egg) to 1⋅00 (meat/poultry/fish and dairy). The FFQ is valid to assess and rank individuals in terms of intake of most food groups according to high and low intake categories.

## Introduction

Nutritional epidemiology, principally dietary intake assessment, plays an essential role in chronic disease studies and general public health concerns^(1–3)^. Besides environmental and lifestyle factors, improving dietary habits is a major target in the prevention of non-communicable diseases (NCDs), such as cancer, cardiovascular diseases, diabetes and chronic kidney diseases^(4)^. In low-income and low-middle income countries, there has been a rapid rise in NCDs, almost half of global premature NCD deaths occur in these settings^(5)^. More research is needed to explore the potential causes of this rising burden and to enable governments to develop targeted preventative policies.

In Ethiopia, although data relating to dietary quality remain sparse, a finding from the Global Burden of Disease Study (GBD) estimated that the number of deaths attributable to dietary factors was 60 402 in 2016^(6)^. The proportion of NCD deaths associated with low fruit consumption slightly increased from 11⋅3 % in 1990 to 2016 11⋅9 %. During this time period, the rate of burden of disease associated with poor diet (diet low in fruits, vegetables, whole grain, nuts and seeds, milk, fibre, calcium, seafood ω-3, polyunsaturated fatty acids; diet high in red and processed meat, sugar-sweetened beverages, trans fatty acids and sodium) slightly decreased; however, the contribution of poor diet to NCDs remained stable^(6)^.

In NCDs, the conceptual exposure is long-term diet. The Food Frequency Questionnaire (FFQ) is a suitable method for assessing habitual dietary intake over longer reference periods. Food frequency questionnaires (FFQs) ask respondents about their usual frequency of consumption of each food during a specified time period^(7)^. Compared with other dietary assessment methods, such as short-term recall and diet records, FFQs are easier to administer, place less burden on respondents, have a relatively low cost and provide a rapid estimate. This makes the FFQ more feasible and better suited for measuring long-term dietary intake for most epidemiological studies and large cohort studies^(8)^.

The interpretation of results from diet-disease studies that use the FFQ is often difficult unless it has been adapted and validated in a population reasonably similar to that being investigated^(9)^. Incorrect information may give rise to false associations between dietary factors and diseases. The null association could also be attributed to a lack of variation in the dietary exposure in the study population or the inability of the tool to find out existing differences in the diet. Therefore, it is important to assess the degree to which the questionnaire measures the aspect of a diet for which it has been designed^(9,10)^.

Validation studies compare one method with another method that is judged to be superior^(11)^. Among the available and feasible comparison methods to validate the FFQ, diet records represent an optimal comparison method, as they have the least correlated error with the FFQ^(9)^. However, when the co-operation or literacy of study subjects is limited, 24-h recall (24-HR) is more appropriate^(9,12)^. Approximately 75 % of validation studies of the FFQ are validated against repeated 24-HRs, preferred for their accuracy to capture daily consumption of a varied diet and for their relatively easy administration and analysis compared with other dietary questionnaires^(12)^.

FFQs may need to be developed and validated specifically for each region in order to be culturally sensitive and to correspond to the prevailing food culture. If the form of the questionnaire is not reasonably appropriate for the cultural background of the study population, developing a new questionnaire is the best approach^(9)^. To our knowledge, there is no validated standard FFQ in Ethiopia that can help to assess dietary intake/habits of adults. Therefore, this present study aimed to develop a context-specific FFQ for Ethiopian and evaluate the validity against two 24-HRs.

## Methods

### Study design and participants

We validated the FFQ against the average of two 24-HRs. The FFQ was obtained 15 d after the second 24-HR was completed. There was a 15-d interval between the first and second 24-HRs. We used an interactive, multiple-pass 24-HR method adapted and validated for use in developing countries^(13)^. We conducted this present study among 120 randomly selected Ethiopian adults aged 20–65 years in Butajira Health and Demographic Surveillance Site (HDSS), from March to April 2019. We employed simple random sampling to identify study participants. Households with adults aged 20–65 years were filtered out from the HDSS data registry to form a sampling frame. From this frame, we randomly selected 120 households with adults aged 20–65 years. We visited all randomly selected households with adults aged 20–65 years with support from the health extension workers, local guides and study supervisors. To be included in the study, the participants had to complete an FFQ and two 24-HRs and have fewer than 10 % of their FFQ items missing^(14)^. After an explanation about the purpose and related procedures of the study, verbal informed consent was obtained from the study participants.

### Development of the FFQ

**Fig. 1.**
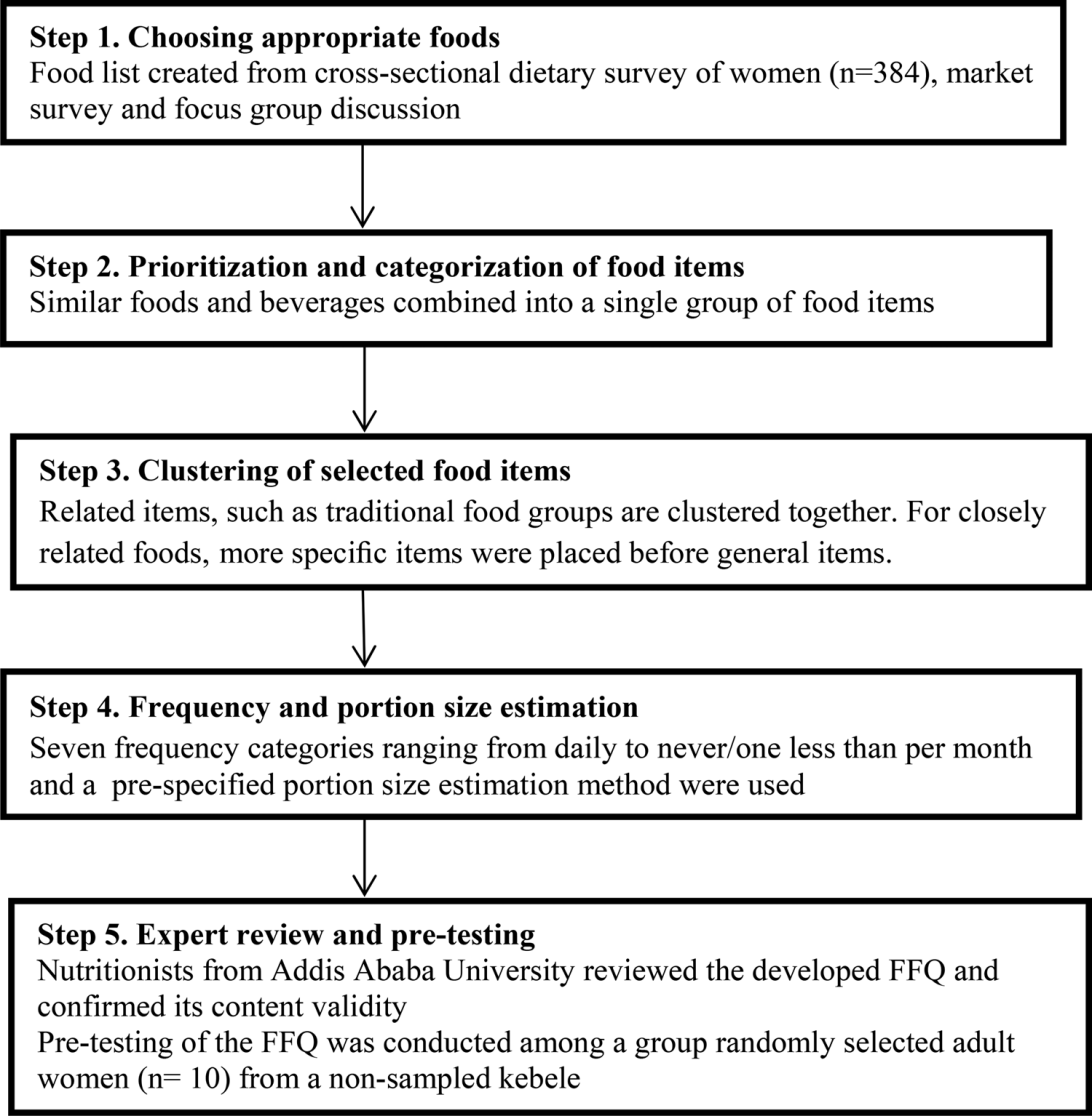
Food frequency development.

Fig. 1 shows the process of FFQ development. We followed five steps to develop the FFQ: choosing appropriate foods, prioritisation and categorisation of food items, assembling a list of selected foods, frequency and portion size, and expert review and pre-testing.

First, we obtained information on dietary intake from an unpublished cross-sectional dietary survey of women (*n* 384) living in rural and urban households of Butajira Health and Demographic Surveillance Site (HDSS), in Southern Nations and Nationalities and Peoples Regional States (SNNPR) from 2018 to 2019. Information on dietary intake was collected using a single multiple-pass 24-HR technique with women in their own homes. The survey was part of a mother–child cohort study (BUNMAP) in Butajera, Southern Ethiopia looking at ‘The economic, psychological, safety and quality aspects of food and nutrition and the effects on pregnancy outcomes, child growth and development’^(15)^.

We undertook market and mini-market visits on non-consecutive days to identify common brand names and foods that could be relevant, and these were added accordingly. In addition, we conducted a focus group discussion in the Butajira district 2 weeks before the interviews began. This was organised by the principal investigator and field supervisors. The focus group consisted of six women who were interviewed about foods consumed in the area. The women were recruited by health extension workers, local guides and study supervisors. The women came from urban and rural areas of Butajira. They were selected purposively. The aim of the focus group discussion was to probe and discuss food items in the study area in order to obtain a comprehensive list of food types, including ingredients used and methods of preparation.

Secondly, we combined similar foods and beverages into a single group of food items^(16)^. Thirdly, we clustered the related food items together. To facilitate dietary reporting, food groupings should fit within respondents’ conceptual framework. We clustered related items together, such as traditional food groups. For closely related foods, we placed a traditional diet before general items. We used the results of our focus group discussion to help construct lists of culturally specific questions and to provide information about which foods should be grouped together.

Fourthly, before data collection began, we evaluated the frequency of intake based on the usual intake over 1 month. We included seven frequency categories ranging from daily to never/less than once per month. Three women were involved in the cooking process and portion size estimations. Those three women are familiar with Ethiopian cuisine. They were asked to prepare different food items according to their areas of skill. They used a recipe prepared using the focus group discussion and Ethiopian food composition table as a guide. We assigned a portion size for each food item. We employed a pre-specified portion size estimation method for estimating portion sizes when using the FFQ using local household units such as bowl, plate, spoons of different sizes (tablespoon and teaspoon), coffee cups, tea cups and water glasses, as well as using photographs. The data for preparing a pre-specified portion size were based on data obtained from food lists created in step one, focus group discussion and local markets and shops visits. To determine the weight of the food items, we made commonly consumed dishes in the Ethiopian Public Health Institute laboratory. We took measurements with an electronic seca scale, and the average of three measurements was used. We gave codes for different prepared portions. To help standardise participants understanding, the interviewer prepared photographs for each measurement and showed them to the participants. When we say we used a pre-specified portion size estimation approach, we mean we asked an additional question regarding the usual portion size for each food. We used household utensils and salted replicas of foods to define a medium, big or small portion size and asked the individuals to classify their usual portion as small, medium or large. During the preparation of portion sizes, we took into account the disparities in portion sizes between men and women. During our focus group discussion with women, we gathered information on portion sizes (medium, small and large). We asked women what kind of utensils they used to serve their husband's food. In that particular case, women are the ones who prepare food for men.

Finally, experts reviewed the newly developed FFQ (nutritionist from Addis Ababa University) to confirm its content validity. We discussed the food list extensively to ensure that wall relevant food items were included. Pre-testing of the FFQ was conducted among a group of 10 randomly selected adult women from a non-sampled kebele who were comparable to the study participants. They were some minor changes in portion sizes used to describe fast foods (burgers and pizzas).

The developed FFQ consisted of 89 food and drink items (supplementary material). The food groups include cereals, bread and potatoes, legumes and pulses, roots and tubers, vegetables, fruits, egg, milk and dairy, fish and fish products, meat and poultry, fat and oil, sweets, drinks, and fast foods and pastry.

### Dietary assessment

#### 24-h dietary recall

We used an interactive, multiple-pass 24-HR method adapted and validated for use in developing countries^(13)^. We conducted the two 24-HRs on non-consecutive days. We interviewed on weekdays and weekends to capture variance in intake across various days of the week. Before data collection, we gave rigorous training to data collectors and conducted a pre-test. We recruited three interviewers who had a previous experience in dietary data collection and who were fluent in the local language. Each interview involved a stepwise series of questions.

First, we asked the participants to report everything they had consumed the previous day, including at night. The opening question was: ‘After you got up this morning/yesterday morning, when was the first time that you had something to eat or drink?’ followed by the questions ‘What did you eat or drink at that time?’ and ‘Did you eat or drink anything else at that time?’ The same three questions were repeatedly asked until the participants had recalled all the food and drink items consumed over the specified period. The first pass ended with the questions ‘Can you remember any other times you had something to eat or drink?’ In the second pass, participants were asked to provide additional detailed information about each item of food and drink consumed. This included the name of the food item, where they ate it, brand names, cooking methods, amounts served and the amount consumed. For home-made dishes, participants were asked for the recipes and ingredients.

On the third pass, we used common household utensils such as bowl, plate, spoons of different sizes (tablespoon and teaspoon), coffee cups, tea cups and water glasses to improve the memory of the respondents and to assist in completing the recall. To estimate portion size, each participant was asked to put the amount of food equivalent to that eaten on weighing scales. The data collectors measured the weight of the food consumed and recorded it. The final pass reviewed all previously recalled information to confirm the accuracy of the record. During the final pass, the data collectors asked the participants about food and drink items not mentioned that were considered to be easy to forget, such as snacks, fruits, water and juices^(17)^.

#### Food Frequency Questionnaire

We evaluated the frequency of intake based on the usual intake over the previous month. We included seven frequency categories ranging from daily to never/less than once per month. Each food item was assigned a pre-specified portion size.

### Calculation of daily food and nutrient intakes

We used the Ethiopian food composition table to derive nutrient and energy estimates from the dietary data^(16)^. The names of food and drink, their description, cooking methods and amounts from both the 24-HRs and the FFQ, were coded and entered into NutriSurvey2007. The FFQ consisted of 89 food and drink items. We organised the food lists into fourteen food groups on the basis of prior information. We calculated food estimates from the FFQ using the product sum method. We converted the average frequency of food intake per week and month of the FFQ to a daily intake value (e.g. frequency of two to three times per month = 2⋅5/3⋅5 times per day). Once the frequency of consumption per day was calculated, we computed the daily food intake using the product sum method. Daily food intake = ∑ (reported consumption frequency of the food item, converted to times per day) * (portion size consumed of that food)^(18)^.

### Statistical test of validity

We checked both the FFQ and 24-HR data for completeness and potential errors. We entered socio-demographic data into Epi-Data version 3.1 and exported to STATA version 15 for further processing and analysis. Out of 120 study participants, 118 (98⋅3 %) of participants completed the first 24-HR, 116 (98⋅6 %) completed the second 24-HR and 115 (95⋅8 %) participants completed both the 24-HRs and the FFQ.

We checked the normality of the average intake of nutrient and food groups using the Shapiro–Wilk normality test and visualised using Q–Q plots. We used parametric tests for normally distributed variables, while non-parametric tests were used for most of the variables as the distributions significantly deviated from normality. Those which fulfilled the assumption of normality were described using mean with standard deviation (sd) and those which do not use median with inter-quartile range (IQR). We evaluated the performance of the FFQ against two 24-HRs using several statistical tests.

First, to compare median daily food intake obtained from the averages of the two 24-HRs and the FFQ, we used the Wilcoxon signed-rank test. To evaluate the agreement between the two methods, we compared the mean daily energy and macronutrient intake obtained from the averages of the two 24-HRs and the FFQ using the paired *t*-test.

Secondly, to measure the strength and direction of the correlation between the two methods, we computed the crude Pearson correlation for normally distributed variables, whereas crude Spearman's *ρ* for those not normally distributed. The cut-off points used for correlation coefficient are as follows: <0⋅20 as low correlation, 0⋅20–0⋅49 as moderate correlation and ≥0⋅50 as high correlation^(11)^.

We calculated the de-attenuated correlations to remove the within-person variability found in the 24-HRs using the following formula:
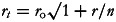
where *r_t_* is the corrected correlation between energy/nutrient/food group derived from the FFQ and the 24-HRs, *r*_o_ is the observed correlation, *r* is the ratio of estimated within-person and between-person variation in energy/nutrient/food group intake derived from the 24-HRs and *n* is the number of replicated recalls (*n* 2)^(9)^.

We also adjusted for total energy intake by using the nutrient density method. For macronutrients (protein, fat and carbohydrate), nutrient densities are expressed as a proportion of energy (i.e. %kcal from protein, %kcal from fat and %kcal from carbohydrate). For micronutrients, nutrient density is expressed as intake (in appropriate units)/1000 kcal^(9)^.

Third, for both the test and reference methods, subjects were divided into categories relating to the distribution of their dietary intake quartiles. A comparison of the subjects’ categories showed the percentage of participants correctly classified in the same category and the percentage misclassified in the opposite category (opposite quartile). The result permitted an assessment of the proportion of subjects who are classified correctly. We used a weighted *κ* statistic to account for both the correctly classified percentage and the expected participant proportion classified by chance. The cut-off points used for weighted *κ* statistics are as follows: <0⋅20 as low *κ* (poor outcome), 0⋅20–0⋅50 as moderate *κ* (acceptable outcome) and ≥0⋅50 as high *κ* (good outcome)^(11)^. Finally, we used a Bland and Altman plot for assessing limits of agreement between the two methods. The Bland–Altman method is preferable to compare two measurements, each of which produced some error in their measures^(19)^.

## Results

### Study participant characteristics

Table 1 shows the socio-demographic characteristics of the study participants. Of the 120 participants, 115 (95⋅8 %) completed both 24-HRs and the FFQ. A total of 105 study participants were included in the final analysis, of which 43 (41 %) were men and 62 (59 %) women. Those excluded are people who made mistakes in their FFQ. The mean age of participants was 31⋅9 years (sd: 9⋅2): 33⋅3 % of them had primary education, and 43 (41 %) were housewives.

**Table 1.** Socio-demographic characteristics of study participants (*n* 105)

Characteristics of participants	Frequency	%
Sex		
Male	43	41
Female	62	59
Age category		
20–29	49	46⋅7
30–39	35	33⋅3
40–49	13	12⋅4
50–65	8	7⋅6
Education		
Primary	35	33⋅3
Secondary	21	20
College/university	10	9⋅5
No formal education	39	37⋅2
Occupation		
Farmer and housewife	5	4⋅8
Housewife	43	41
Employee/private	3	2⋅9
Merchant	25	23⋅8
Daily labourer	8	7⋅6
Unemployed	8	7⋅6

### Relative validity analysis

Table 2 shows the mean (sd), median, and 25th and 75th percentiles daily nutrient intakes estimated by the average of two 24-HRs and the FFQ. The mean energy and macronutrient intake obtained from the FFQ were significantly higher than the average of the two 24-HRs. The mean difference for energy was 368 (95 % CI: 259⋅0, 476⋅1). The mean difference for total fat intake was 4⋅1 (95 % CI: 2⋅5, 5⋅7). Similarly, a significant median difference was found in micronutrient intake between the two measures. The median difference ranged from 0⋅09 mg/day for vitamin B2 to 391⋅8 μg RAE for vitamin A intake.

**Table 2.** Mean (sd), median, and 25th and 75th percentiles of daily energy and nutrient intakes estimated by the average of two 24-h dietary recalls and the FFQ

Energy and nutrient	Average of 24-h dietary recalls		FFQ		Paired *t*-test	
	Mean	sd	Mean	sd		*P*-value
Energy (kcal)	1449⋅50	421⋅60	1817⋅10	482⋅30	367⋅60*	0⋅000
Protein (g)	39⋅40	12⋅20	49⋅90	12⋅50	10⋅50*	0⋅000
Total fat (g)	17⋅20	5⋅70	21⋅30	6⋅10	4⋅10*	0⋅000
Carbohydrate (g)	297⋅60	92⋅30	375⋅20	109⋅30	77⋅60*	0⋅000
	Median	IQR (25 %, 75 %)	Median	IQR (25 %, 75 %)	Wilcoxon signed-rank test	
Calcium (mg)	463⋅10	337⋅60, 587⋅90	684⋅40	518⋅00, 796⋅80	0⋅000**	
Iron (mg)	56⋅00	41⋅90, 83⋅70	67⋅40	54⋅30, 86⋅80	0⋅009**	
Vitamin A (μg RAE)	259⋅30	51⋅50, 581⋅40	651⋅10	295⋅90, 976⋅60	0⋅000**	
Vitamin B1 (mg)	0⋅70	0⋅49, 0⋅98	0⋅81	0⋅54, 1⋅17	0⋅002**	
Vitamin B2 (mg)	0⋅70	0⋅54, 0⋅84	0⋅79	0⋅64, 0⋅94	0⋅002**	

FFQ, Food Frequency Questionnaire; IQR, Inter-quartile range.

* *P* ≤ 0⋅05; **P < 0⋅01.

Table 3 presents the results of correlations between nutrient intake obtained from the average of two 24-HRs and the FFQ. The Crude Pearson correlation varied from 0⋅05 (total fat) to 0⋅32 (carbohydrate). Except for total fat, the correlations were statistically significant. Energy adjusted correlation for macronutrients varied from 0⋅20 (protein) to 0⋅51 (carbohydrate). Energy adjusted correlation for micronutrients varied from 0⋅12 (calcium and iron) to 0⋅39 (vitamin B1). Spearman's correlation (*ρ*) obtained for micronutrients ranged from 0⋅1 (calcium) to 0⋅49 (vitamin B1). A statistically significant correlation was obtained for vitamin A (*P* < 0⋅05) and vitamin B1 (*P* < 0⋅05). De-attenuation improved correlation for all nutrients. The de-attenuated correlation ranged from 0⋅10 (total fat) to 0⋅80 (vitamin A).

**Table 3.** Correlations of daily energy and nutrient intakes when comparing the FFQ to the average of two 24-h dietary recalls

Macronutrients	Pearson's correlation (95 % CI)			Bland–Altman statistics		
		Energy adjusted correlation (95 % CI)	De-attenuated correlation	Mean difference (95 % CI)		95 % limit of agreement
Energy (kcal)	0⋅24* (0⋅05,0⋅41)	–	0⋅38	367⋅6 (259⋅0, 476⋅1)*		−731⋅9, 1467⋅1
Protein (g)	0⋅22* (0⋅03, 0⋅36)	0⋅20* (0⋅006, 0⋅375)	0⋅36	10⋅5 (7⋅6, 13⋅5)*		−19⋅7, 40⋅8
Total fat (g)	0⋅05 (−0⋅14, 0⋅24)	0⋅26* (0⋅076, 0⋅433	0⋅10	4⋅4 (2⋅5, 5⋅7)*		−13⋅7, 22⋅5
Carbohydrate (g)	0⋅32* (0⋅14, 0⋅48)	0⋅51* (0⋅36 to 0⋅64)	0⋅51	77⋅6 (54⋅5, 100⋅6)*		−155⋅9, 311
Micronutrients	Spearman's correlation (95 % CI)	Energy adjusted correlation (95 % CI)	De-attenuated correlation	Bland and Altman statistics for log-transformed data		
				Mean difference (95 % CI)	95 % limit of agreement	
Calcium (mg)	0⋅10 (−0⋅09, 0⋅29)	0⋅12 (−0⋅06 to 0⋅32)	0⋅20	0⋅15 (0⋅11, 0⋅18)*	−0⋅26, 0⋅55	
Iron (mg)	0⋅12 (−0⋅07, 0⋅30)	0⋅12 (−0⋅07 to 0⋅31)	0⋅23	0⋅06 (0⋅007, 0⋅11)*	−0⋅45, 0⋅61	
Vitamin A (μg RAE)	0⋅45* (0⋅28, 0⋅59)	0⋅37* (0⋅19 to 0⋅52)	0⋅80	0⋅5 (0⋅34, 0⋅66)*	−1⋅1, 2⋅1	
Vitamin B1 (mg)	0⋅49* (0⋅33, 0⋅62)	0⋅39* (0⋅22 to 0⋅54)	0⋅75	0⋅1 (0⋅02, 0⋅11)*	−0⋅4, 0⋅5	
Vitamin B2 (mg)	0⋅17 (−0⋅02, 0⋅35)	0⋅07 (−0⋅12 to 0⋅26)	0⋅35	0⋅05 (0⋅01, 0⋅09)*	−0⋅41, 0⋅51	

CI, Confidence interval.

* *P* ≤ 0⋅05.

Table 4 shows cross-classification and weighted *κ* statistics of daily intakes of energy, nutrients and food group in quartiles assessed with the average of the two 24-HRs and the FFQ. The proportion of individuals classified by the FFQ and the average of two 24-HRs into the same quartile ranged from 13⋅4 % for total fat to 38⋅1 % for vitamin A. However, the proportion classified into opposite quartiles varied from 3⋅8 % (vitamin B1) to 23⋅8 % (total fat). Weighted *κ* values ranged from −0⋅04 (total fat) to 0⋅18 (vitamin A).

**Table 4.** Cross-classification and weighted *κ* statistics of daily energy and nutrient intakes of food group in quartiles as assessed with the average of two 24-hour dietary recalls and the FFQ

Energy and nutrient	Cross-classification		*κ* statistics
	% in the same quartile of individuals	% in the opposite quartile of individuals	*κ* value
Energy (kcal)	34⋅3	8⋅6	0⋅13
Protein (g)	33⋅4	8⋅6	0⋅11
Total fat (g)	13⋅4	23⋅8	−0⋅04
Carbohydrate (g)	34⋅3	8⋅6	0⋅12
Calcium (mg)	25⋅7	7⋅6	0⋅09
Iron (mg)	28⋅6	9⋅5	0⋅05
Vitamin A (μg RAE)	38⋅1	5⋅7	0⋅18
Vitamin B1 (mg)	33⋅3	3⋅8	0⋅11
Vitamin B2 (mg)	33⋅3	8⋅6	0⋅11

Table 5 presents median, and 25th and 75th percentiles of daily food group intakes estimated by the average of two 24-HRs and the FFQ. Both methods provide similar median intake estimates for fruits, eggs, meat/poultry/fish and daily products. For roots and tubers, the 24-HRs show a higher estimate of median intake. The FFQ provides a higher estimate of median vegetable intake. A statistically significant median difference was only observed for roots and tubers and vegetable intake.

**Table 5.** Mean (sd), median, and 25th and 75th percentiles daily food group intakes estimated by the average of two 24-hour dietary recalls and the FFQ

Food group	Average of 24-h dietary recalls		FFQ		
	Median	(25 %, 75 %)	Median	(25 %, 75 %)	*P*-value
Cereals (g)	710	(548⋅5, 817)	648	(520⋅9, 852)	0⋅997
Legumes (g)	94⋅5	(0, 145⋅5)	93	(21, 134⋅9)	0⋅347
Roots and tubers (g)	24⋅5	(0, 45)	11⋅2	(0, 31⋅3)	0⋅013*
Vegetables (g)	79⋅5	(23⋅5, 156)	109	(45⋅3, 159)	0⋅048*
Fruits (g)	0⋅0	0⋅0	0⋅0	(0, 15⋅4)	0⋅367
Eggs (g)	0⋅0	0⋅0	0⋅0	0⋅0	0⋅000**
Dairy products (g)	0⋅0	0⋅0	0⋅0	(0, 4⋅9)	0⋅087
Meat/poultry/fish (g)	0⋅0	0⋅0	0⋅0	0⋅0	0⋅068
Beverages (g)	243	(152, 334)	230⋅4	(183, 320⋅6)	0⋅971

Wilcoxon signed-rank test: *P ≤ 0⋅05; **P < 0⋅01.

Table 6 shows the correlations between food group intake obtained from the average of the two 24-HRs and the FFQ. The crude Spearman correlation ranged from 0⋅12 for eggs to 0⋅78 for legumes. Greater than 0⋅5 correlations were observed for legumes (*r* 0⋅78). Correlation (0⋅2–0⋅49) were observed for cereals (*r* 0⋅33), meat/poultry/fish (*r* 0⋅47), fruits (*r* 0⋅46), dairy products (*r* 0⋅45), roots and tubers (*r* 0⋅34), vegetables (*r* 0⋅3) and beverages (*r* 0⋅2). Correlation was low (<0⋅2) for egg (*r* 0⋅12). De-attenuation improved correlation for all food groups. The de-attenuated correlation ranged from 0⋅24 (egg) to 1⋅00 (meat/poultry/fish and dairy). Greater than 0⋅5 correlations were observed for all food groups except eggs and beverages.

**Table 6.** Correlations of food group intakes when comparing the FFQ to the average of two 24-Hour dietary recalls

Food groups	Spearman's correlation (95 % CI)		Bland–Altman statistics			
		De-attenuated correlation	Mean difference (95 % CI)		95 % limit of agreement	
Cereals	0⋅33* (0⋅15, 0⋅49)	0⋅62	9⋅9 (−41⋅9, 61⋅8)		535⋅3, −515⋅5	
Legumes	0⋅79* (0⋅71, 0⋅85)	1⋅60	2⋅6 (−8⋅9, 14⋅1)		−113⋅8, 118⋅9	
Vegetables	0⋅33* (0⋅15, 0⋅49)	0⋅62	7⋅1 (−15⋅7, 29⋅8)		−223⋅7, 237⋅8	
Beverages	0⋅20* (0⋅01,0⋅38)	0⋅40	2⋅9 (−30⋅9, 36⋅6)		−339, 344⋅7	
	Spearman's correlation (95 % CI)	De-attenuated correlation	Bland and Altman statistics		Bland and Altman statistics for log-transformed data	
			Mean difference (95 % CI)	95 % limit of agreement	Mean difference (95 % CI)	95 % limit of agreement
Roots and tubers	0⋅34* (0⋅16, 0⋅45)	0⋅55	16⋅2 (6⋅2,26⋅3)*	−87⋅5, 120⋅1	−0⋅04 (−0⋅23,0⋅15)	−1⋅96, 1⋅88
Fruits	0⋅46* (0⋅23, 0⋅56)	0⋅90	7⋅9 (1⋅4, 14⋅6)*	−60⋅2, 76⋅2	0⋅15 (0⋅01,0⋅29)*	−1⋅25, 1⋅55
Eggs	0⋅12 (−0⋅07, 0⋅30)	0⋅24	−2⋅1 (−3⋅4,0⋅8)*	−15⋅9, 11⋅7	0⋅19 (0⋅11,0⋅27)*	−0⋅64, 1⋅03
Dairy products	0⋅45* (0⋅29, 0⋅59)	1⋅00	6⋅2 (1⋅9, 10⋅4)*	−37⋅8, 50⋅1	0⋅04 (−0⋅07, 0⋅14)	−1⋅03, 1⋅1
Meat/poultry/fish	0⋅47* (0⋅31, 0⋅61)	1⋅00	3⋅8 (−0⋅8, 8⋅4)	−43⋅2, 50⋅8	0⋅06 (−0⋅01, 0⋅13)	−0⋅68, 0⋅8

* *P* ≤ 0⋅05.

Table 7 shows cross-classification and weighted *κ* statistics of daily intake of food groups in quartiles as assessed with an average of the two 24-HRs and the FFQ. The highest correct classification into the same quartile was observed for cereals and legumes – i.e. 50⋅5 and 51⋅4 %, respectively. For the other food groups, the classification into the same quartile ranged from 30⋅5 % (beverages) to 40 % (roots and tubers). Oppositely, classified individuals ranged from 1 % (cereals) to 11⋅4 % (beverages). No gross misclassification was observed for the intake of legumes. Weighted *κ* values ranged from 0⋅07 (beverages) to 0⋅35 (legumes).

**Table 7.** Cross-classification and weighted *κ* statistics of daily intakes of food group in quartiles as assessed with the average of two 24-h dietary recalls and the FFQ

Food groups	Cross-classification		*κ* statistics
	% in the same quartile of individuals	% in the opposite quartile of individuals	*κ* value
Cereals	50⋅5	1	0⋅32
Legumes	51⋅4	0	0⋅35
Roots and tubers	40	7⋅6	0⋅18
Vegetables	38⋅1	6⋅7	0⋅17
Beverages	30⋅5	11⋅4	0⋅07

**Fig. 2.**
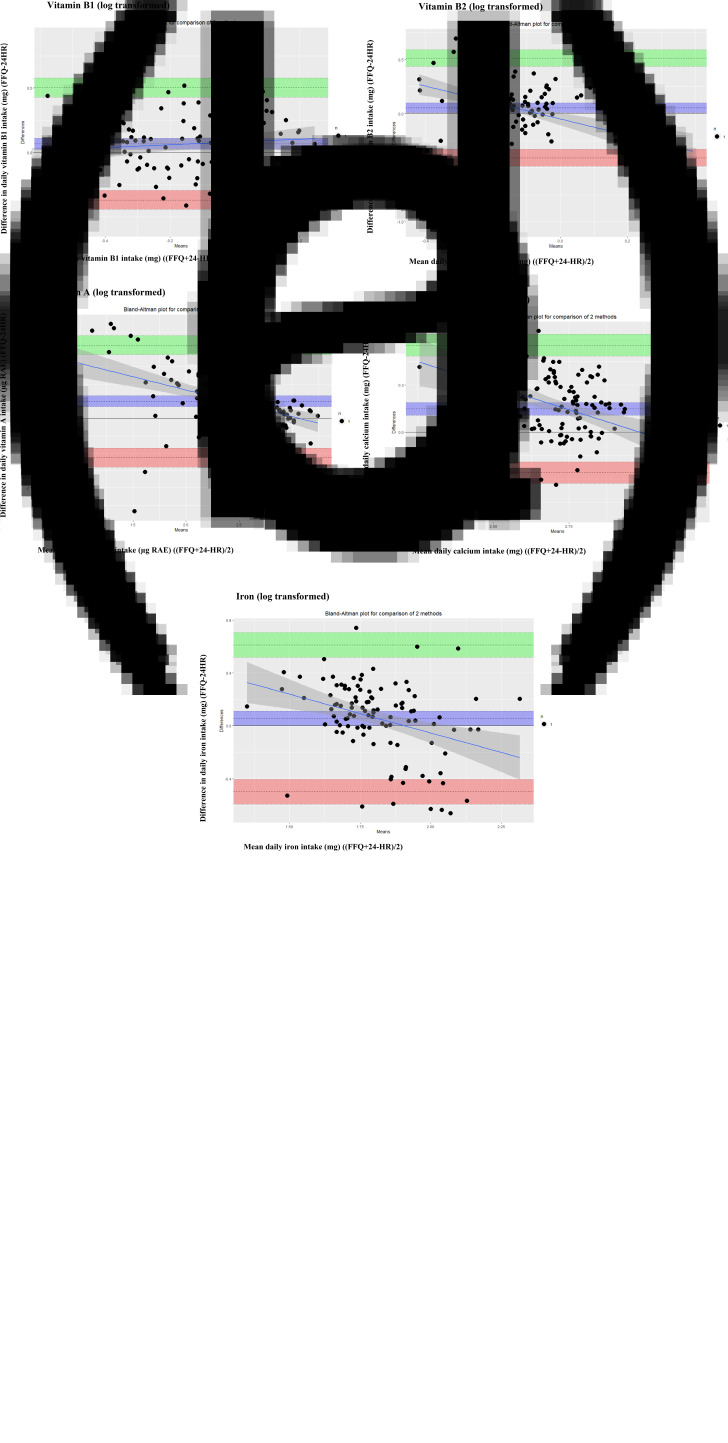
Bland–Altman analysis plot of (a) energy, (b) protein, (c) carbohydrate, (d) total fat, (e) vitamin B1, (f) vitamin A, (g) vitamin B2, (h) calcium and (i) iron as predicted by the FFQ and the average of two 24-h dietary recalls.

Fig. 2 presents the Bland–Altman plots for energy, protein, carbohydrate, total fat, vitamin B1, vitamin A, vitamin B2, calcium and iron. The Bland–Altman plot was used to evaluate the agreement between the FFQ and the 24-HRs by plotting the difference between the two methods versus the average of the two methods for each nutrient and calculating the limits of agreement and their corresponding 95 % CI. The FFQ overestimated energy and macronutrient intake. Except for total fat intake, increased variability of data points was observed for all nutrients both at low, average and high values (wider limits of agreement). Some outliers were observed for energy and macronutrients. Since differences in nutrient intake were associated with the mean measurement, data related to the micronutrient intake were log-transformed for Bland and Altman statistics. The results indicate a trend, as the FFQ consistently overestimated vitamin A and iron intake at a lower value.

**Fig. 3.**
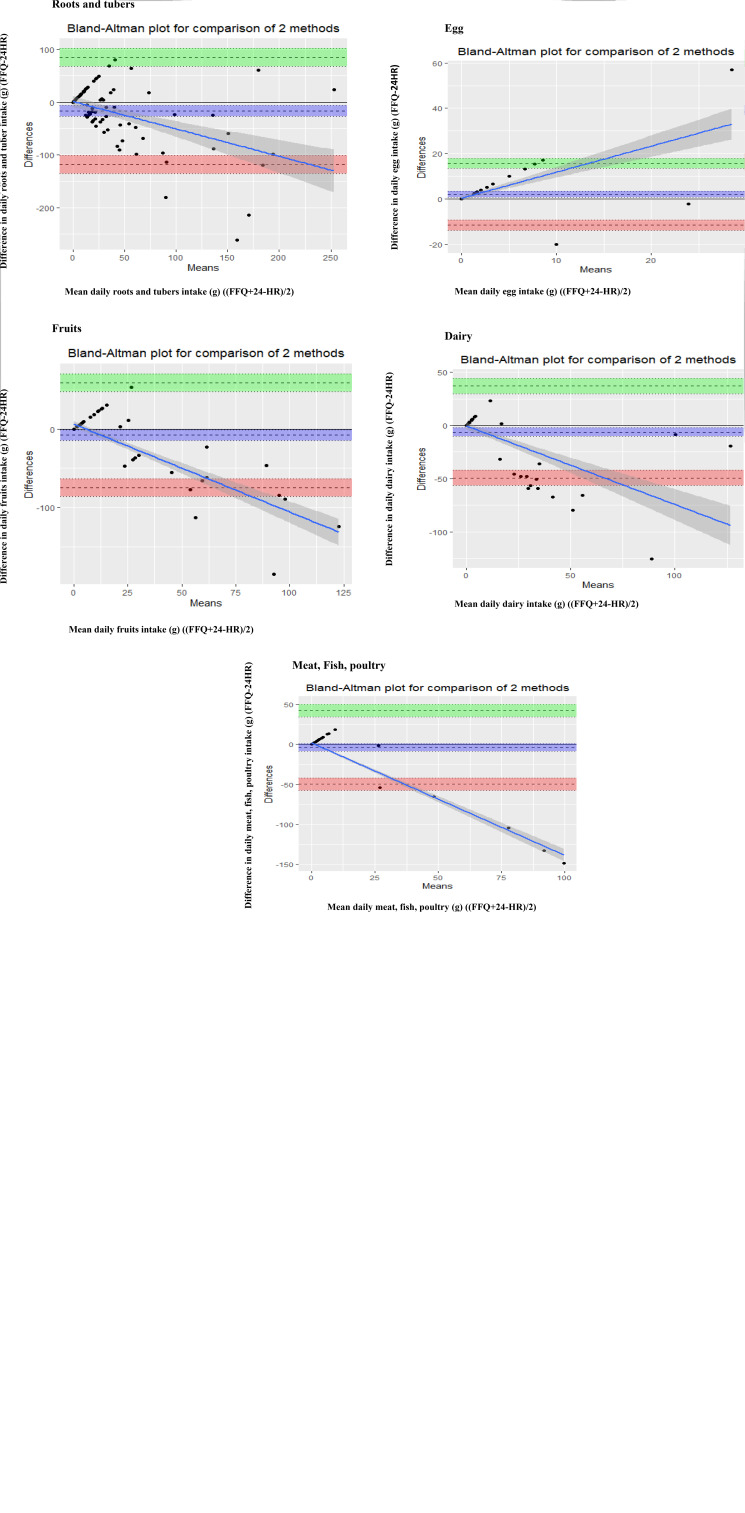
Bland–Altman analysis plot of (a) legumes, (b) cereals, (c) vegetables, (d) beverages, (e) roots and tubers, (f) fruits, (g) egg, (h) dairy product and (i) meat/poultry/fish as predicted by the FFQ and the average of two 24-h dietary recalls.

Fig. 3 shows the Bland–Altman plots for legumes, cereals, vegetables, beverages, roots and tubers, fruits, egg, dairy product and meat/poultry/fish. Data relating to roots and tubers were log-transformed for Bland and Altman statistics. A systematic trend of overestimation for roots and tubers and underestimation of beverage intakes at higher values was observed when we used the FFQ. The majority of the data points are with in the 95 % of limits of agreement for almost all food groups. A wide limit of agreement was observed for roots and tubers.

## Discussion

In this present study, we developed and validated an FFQ to assess the food and nutrient intake of adults in Ethiopia. We observed a higher intake of energy and nutrients when the FFQ was used compared with the average of the two 24-HRs. Bland–Altman plots show an overestimation of energy and macronutrients (carbohydrate, protein and fat) for various data points. We found a low to moderate level of agreement (correlation coefficients) for energy and nutrient intakes between the two methods.

We found that the FFQ overestimated energy and nutrient intakes relative to the average of the two 24HRs. Overestimation is a common issue reported in various validation studies^(10–15)^. The mean percent difference in the present study was poor (>10 %)^(11)^ for intakes of energy (15⋅5 %), protein (39 %), carbohydrate (36⋅6 %) and total fat (44⋅5 %) compared with other validation studies conducted using 24-HRs as a reference method^(14,17,20–22)^. Overestimation can be attributed to the subject's tendency to overestimate their actual intake when they are asked to recall the frequency of a large number of foods consumed in an FFQ. Furthermore, difficulty in conceptualising the assigned portion sizes and difficulties in reporting the frequencies of usual intake could be a contributing factor^(22)^. It may have also occurred as a result of purposeful over-reporting of food consumption by subjects^(13)^. The use of shorter questionnaires and advances in portion size estimation techniques are suggested to address overestimation of intake by FFQs.

The present study found moderate crude correlations (0⋅2–0⋅49) between the average of the two 24-HRs and the FFQ for energy (*r* 0⋅24), protein (*r* 0⋅22) and carbohydrate (*r* 0⋅32) and crude low correlation (<0⋅2) for fat (*r* 0⋅05), after adjusting for energy the correlation for total fat increased (*r* 0⋅26). The moderate energy-adjusted correlation found between the two methods for macronutrient intake is comparable with other previous validation studies^(17,20,21,23)^. However, our finding was lower than those reported by other studies using 24-HRs as a reference method^(17,20,21)^. The observed moderate energy-adjusted correlation could be interpreted as a result of using only a 2-d 24-HRs as a reference method. However, after correcting for within-person variability, the correlation for all nutrients improved. Moderate to good de-attenuated correlations were reported for the majority of nutrients. The moderate to good de-attenuated correlation observed was comparable to other FFQ validation studies^(17,22,24,25)^.

The lower crude correlation for iron and vitamin intake observed in the present study is not uncommon in FFQ validation studies^(14,17,20,22,26)^. A meta-analysis of FFQ validation studies showed that pooled crude correlation coefficients of nutrient intake (total fat, protein, carbohydrate, alcohol, calcium, iron and vitamins) were lower for the FFQ validated against 24-HRs rather than food records^(27)^. The possible reason for a low correlation for the vitamin intake is that vitamin intake tends to vary greatly from day to day, as many vitamins are found in only a small selection of foods^(17)^. A study reported that the number of days required to ensure specified (i.e. 0⋅75, 0⋅80, 0⋅85, 0⋅90 and 0⋅95) correlation coefficients between observed and usual (‘true’) mean intakes of energy and nutrients by food records was large^(28)^.

We observed a moderate to good crude correlation for almost all food groups. This is in agreement with previous validation studies assessing food group intake^(17,20,29,30)^. The good correlation found for vegetable intake in the present study is higher than those reported by other validation studies^(17,20,21)^. This may have occurred because of ease of quantifying vegetable intake, as they are often consumed independently in Butajira. The lower correlation of egg intake in the present study, in contrast to other studies^(14,20)^, may have occurred by chance of not having consumed eggs on the days on which the 24-HRs were conducted, since eggs are consumed once or twice per week in Ethiopia. De-attenuation improved the correlation for most food groups; the highest correlation was found for dairy products (*r* 1⋅00) and meat/poultry/fish (*r* 1⋅00) and the lowest for eggs (0⋅24).

The Bland–Altman plot showed a moderate agreement between the two methods for energy and macronutrients. No trend was observed across energy and macronutrient intakes. A comparable study similarly showed a moderate level of agreement with no persistent trend across intake levels using a Bland–Altman plot^(21,24)^. However, ranges for limits of agreement were relatively wide, differing from another study^(22)^. The observed wide limits of agreements between the FFQ and reference methods are common and highlight the limitations of the FFQ in assessing absolute nutrient intake due to wide variability in how the FFQ measures energy and macronutrient intakes relative to the average of the two 24-HRs^(10)^.

A tendency towards a poorer agreement in vitamin A and iron intake between methods was observed with lower levels of intake as shown by the Bland–Altman plot. This poor agreement in iron intake is also reported in another validation study^(18)^. As indicated by a Bland–Altman plot, a systematic mean difference was not observed across the intake levels of cereals, legumes, vegetables and beverages. Most of the data points are found between the 95 % limits of agreement. However, the plot indicated wide limits of agreement which occur as a result of increased variability.

The present study shows that the FFQ did not adequately classify subjects with respect to energy, macronutrients and most of the micronutrients as indicted by cross-classification and weighted *κ* results (percentage of individuals in same quartile < 50, *k* values < 0⋅2)^(11)^. However, the FFQ showed a fair quartile classification agreement for cereals, legumes and roots and tubers (*k* values 0⋅2–0⋅6). This finding is consistent with previous studies which reported cross-classification and *κ* by categorising intake into quartiles^(14,18,20)^. We found lower values for energy and nutrient intakes with respect to those reported by other studies using similar intake categories^(17,21,22,31)^. The misclassification and low *κ* reported in the present study may have occurred due to the insensitivity of the FFQ in classifying individuals into intake categories. The use of a food diary as a reference method may have also increased classification agreement in previous studies. The FFQ showed a fair quartile classification agreement for cereals, legumes and roots and tubers (*k* values 0⋅2–0⋅6). Similarly, other studies reported a fair classification agreement for these particular food groups^(22,29)^. For beverage intake, the present study indicated a misclassification (30⋅5 %) into opposite quartiles supported by low *κ* value (*k* value <0⋅2), showing a poor outcome. Other studies reported a similar finding for beverage intake^(29)^.

The present study has limitations that must be acknowledged. First, given that we used a 24-HR as our reference method, errors may occur between 24-HRs and the FFQ with regard to the conceptualisation of portion sizes. However, to lessen this effect we used a salted replica of actual foods, pictures and calibrated equipment to estimate portion size. Secondly, we conducted two 24-HRs per participant due to financial and logistic constraints. Two 24-HRs have limitations, particularly for estimating the usual intake of foods not consumed on a daily or regular basis such as egg intake. Therefore, the validity results should be interpreted with caution as low correlations, and large differences may also be due to this issue. We recommended that further research is undertaken to assess this effect. Thirdly, participants may have purposefully over-reported their intake due to social desirability. However, we gave a detailed explanation to the interviewers on how to explain the purpose of the FFQ to participants using role-playing, small group exercises and discussions. Fourthly, we did not administer the FFQ at the onset of the study; therefore, we cannot assess the reproducibility of the instrument. Fifthly, seasonal variation was not taken into account. Therefore, we recommend a further research be undertaken to assess this effect. It should be noted that two 24-HRs have limitations, particularly for estimating energy intake. Therefore, the estimates of energy should be interpreted with caution. We recommend a further research be undertaken to assess underreporting of energy intake.

The strength of the present study was the development of the FFQ based on the latest local dietary survey, focal group discussions, pre-test and expert reviews. Furthermore, the use of comprehensive statistical analysis to assess the validity of the FFQ and the use of interactive, multiple-pass 24-HRs which had been adapted and validated for use in developing countries as our reference method add rigour to the present study.

## Conclusions

The study showed that the FFQ had good validity to capture the intake of cereals, legumes, vegetables and beverages. However, intakes of roots and tubers and beverages should be interpreted with caution. The FFQ is capable of classifying cereals, legumes, roots and tubers, and vegetable intake according to high and low intake categories.

## Supplementary material

For supplementary material accompanying this paper visit http://dx.doi.org/10.1017/jns.2021.94. click here to view supplementary material
