# Supplementary material for: Development and validation of food frequency questionnaire for food and nutrient intakes of adults in Butajira, Southern Ethiopia
Source: J Nutr Sci. 2021 Nov 22;10:e98. doi: 10.1017/jns.2021.94 (PMC8635871; doi:10.1017/jns.2021.94)
Supplement: Supplementary file 1 [file S204867902100094Xsup001.docx]

**Semi quantitative food frequency questionnaire**

**Instruction** –Dear respondent please take few moment to memorize the food and drinks you ate within the last month. These relate to your daily use of food items and also food consumed out of home, e.g. in a restaurant, bar, at work etc. I will say the food items if you consumed the food type you will tell me how often and how much you ate over the last month. When I ask you about your usual portion size you will tell me the average quantity or portion of the food item on the day of consumption, expressed as common household measures such as a ladle, small cup or spoon. We will use pictures to improve your response on the food items consumed.

**If you eat for instance** ‘nech teff enjera’ during four days per week (Monday, Tuesday, Wednesday, and Thursday) you will choose the option that says 4-6 times per week. If you usually consume two (2) full medium sized (eight kurtih) nech teff enjera on these days your answer will be 2 full medium sized enjera or eight kurtih ( if you use kurtis for serving). You will tell me the total enjeras you consumed be it alone or in combination with others. (Examples, bado enjera + enjera be wot + enjera be avocado)

**Remember**: these two full enjeras are the totals of all meals (breakfast, lunch, snacks, and dinner)

**Cereals products, bread and potatoes**

| **Cereals**  Food item | Average consumption over the last month | Average consumption | Usual portion  size (gram) |
| --- | --- | --- | --- |
| **Enjera**  1. Nech teff enjera  2. key teff enjera  3. teff enjera mixed with rice  4. tef enjera mixed with maize  5. maize enjera | Never or < 1 x per month  1 x per month  2- 3 x per month  1 x per week  XX  2-3 x per week  4-6 x per week  Every day | **number of full enjera consumed** (mulu enjera)  1 full enjera = 293g  Number of half enjera consumed  1 half enjera= 153g  number of enjera consumed in kurtih (chopped)  1 kurtih = 97 g | \|  \|  \|  \| \| --- \| --- \| --- \| |
| 6. Enjera firfir | Never or < 1 x per month  1 x per month  2- 3 x per month  1 x per week  XX  2-3 x per week  4-6 x per week  Every day | **Written in Number of portions**  Small portion (91g)  Medium portion (_113g)  Large portion (166g) | \|  \|  \|  \| \| --- \| --- \| --- \| |
| 7. Pasta | Never or < 1 x per month  1 x per month  2- 3 x per month  1 x per week  XX  2-3 x per week  4-6 x per week  Every day | **Large**: Portion of 545 g (full plate)  **Medium** : Portion of 267g (half plate)  **Smal**l: Portion of 186g (1/4 of the plate | \|  \|  \|  \| \| --- \| --- \| --- \| |

| Food item | Average consumption over the last month | Average consumption : **show photos** | Usual portion  size (gram) |
| --- | --- | --- | --- |
| 8. Macaroni | Never or < 1 x per month  1 x per month  2- 3 x per month  1 x per week  XX  2-3 x per week  4-6 x per week  Every day | **Large**: Portion of 587g (full plate)  **Medium** : Portion of 343 g (half plate)  **Smal**l: Portion of 211 g (1/4 of the plate | \|  \|  \|  \| \| --- \| --- \| --- \| |
| 9. Rice | Never or < 1 x per month  1 x per month  2- 3 x per month  1 x per week  XX  2-3 x per week  4-6 x per week  Every day | **Large**: Portion of 420 g (full plate)  **Medium** : Portion of 261 g (half plate)  **Smal**l: Portion of 153 g (1/4 of the plate | \|  \|  \|  \| \| --- \| --- \| --- \| |
| 10. Ye aja kinche | Never or < 1 x per month  1 x per month  2- 3 x per month  1 x per week  XX  2-3 x per week  4-6 x per week  Every day | **Large**: Portion of 545 g (full plate)  **Medium** : Portion of 267g (half plate)  **Smal**l: Portion of 186g (1/4 of the plate | \|  \|  \|  \| \| --- \| --- \| --- \| |
| 11. Nifro (ye sinde) | Never or < 1 x per month  1 x per month  2- 3 x per month  1 x per week  XX  2-3 x per week  4-6 x per week  Every day | **Written in hand/ palm size** (efignih) = 41 g (medium portion) | \|  \|  \|  \| \| --- \| --- \| --- \| |
| Food item | Average consumption over the last month | Average consumption : show photos | Usual portion  size (gram) |
| 12. ye bula genfo | Never or < 1 x per month  1 x per month  2- 3 x per month  1 x per week  XX  2-3 x per week  4-6 x per week  Every day | **Large**: Portion of 684g (full curved plate)  **Medium** : Portion of 407 g (half curved plate)  **Smal**l: Portion of 290 g (1/4 of the plate | \|  \|  \|  \| \| --- \| --- \| --- \| |
| 13. ye gebsi genfo (barley porridge) | Never or < 1 x per month  1 x per month  2- 3 x per month  1 x per week  XX  2-3 x per week  4-6 x per week  Every day | Medium portion/ half plate (267 g)  Large portion/ full plate (426 g) | \|  \|  \|  \| \| --- \| --- \| --- \| |
| **kolo**  14. ye gebsih kolo  15. ye gebsih ena ye shinbira | Never or < 1 x per month  1 x per month  2- 3 x per month  1 x per week  XX  2-3 x per week  4-6 x per week  Every day | **Written in palm size (efignih)**  Medium portion = 30 gm | \|  \|  \|  \| \| --- \| --- \| --- \| |
| 16. Gruel (atmit) | Never or < 1 x per month  1 x per month  2- 3 x per month  1 x per week  XX  2-3 x per week  4-6 x per week  Every day | **Written in number of cups/glasses of gruel show photos**  Size 1 ( ml)  Size 2 ( ml)  Size 3 ( ml) | \|  \|  \|  \| \| --- \| --- \| --- \| |

| **Bread** Food item | Average consumption over the last month | Average consumption | Usual portion size (gram) |
| --- | --- | --- | --- |
| 17. Bread : maize  Ye bokolokita (ambasha) | Never or < 1 x per month  1 x per month  2- 3 x per month  1 x per week  XX  2-3 x per week  4-6 x per week  Every day | Number of large slices (1=390g)  Number of medium slices (1=246 g)  Number of small slices (1=199 g) | \|  \|  \|  \| \| --- \| --- \| --- \| |
| 18. Bread: wheat and maize  ye sindenayebokolokita (ambasha) | Never or < 1 x per month  1 x per month  2- 3 x per month  1 x per week  XX  2-3 x per week  4-6 x per week  Every day | Number of large slices ( 341 g)  Number of medium slices ( 280 g)  Number of small slices ( 258 g) | \|  \|  \|  \| \| --- \| --- \| --- \| |
| 19. Bread white (nech ye sukih/ye furno duket dabo) | Never or < 1 x per month  1 x per month  2- 3 x per month  1 x per week  XX  2-3 x per week  4-6 x per week  Every day | **Ask in monetary value**  Number of large slice (3birr = 285 g)  Number of medium slice (2birr =114 g)  Number of small slice (1/2 the medium = 57 g) | \|  \|  \|  \| \| --- \| --- \| --- \| |
| 20. Sanbusa | Never or < 1 x per month  1 x per month  2- 3 x per month  1 x per week  XX  2-3 x per week  4-6 x per week  Every day | **Ask in monetary value**  Number of large slice (3birr = g)  Number of medium slice (2birr = g) | \|  \|  \|  \| \| --- \| --- \| --- \| |
| **Potatoes**  Food item | Average consumption over the last month | Average consumption | Usual portion  size (gram) |
| 21. Potato boiled | Never or < 1 x per month  1 x per month  2- 3 x per month  1 x per week  XX  2-3 x per week  4-6 x per week  Every day | Number of pieces of average portion boiled potato (1= g) | \|  \|  \|  \| \| --- \| --- \| --- \| |
| 22. Potato fried | Never or < 1 x per month  1 x per month  2- 3 x per month  1 x per week  XX  2-3 x per week  4-6 x per week  Every day | **Large**: Portion of 168g (full plate)  **Medium** : Portion of 109 g (half plate) |  |
| 23. Potato stew | Never or < 1 x per month  1 x per month  2- 3 x per month  1 x per week  XX  2-3 x per week  4-6 x per week  Every day | **Written in Number of ladles**  Small ladle (90 g)  Medium ladle (120 g  Large ladle (308 g) | \|  \|  \|  \| \| --- \| --- \| --- \| |

**Legumes and pulses**

| Food item | Average consumption over the last month | Average consumption | Usual portion  size (gram) |
| --- | --- | --- | --- |
| **Pea stew/Shiro wet**  24. mitin shiro  25. shiro ye aterena bakela  26. shiro ye bekelaen ashimbra  27. ye bakela shiro  28. ye aterena ye guaya shiro | Never or < 1 x per month  1 x per month  2- 3 x per month  1 x per week  XX  2-3 x per week  4-6 x per week  Every day | **Written in Number of ladles**  Small ladle (96 g)  Medium ladle (126g  Large ladle (167g) | \|  \|  \|  \| \| --- \| --- \| --- \| |
| 29. Misirkik wet/ split lentil stew | Never or < 1 x per month  1 x per month  2- 3 x per month  1 x per week  XX  2-3 x per week  4-6 x per week  Every day | **Written in Number of ladles**  Small ladle (62 g)  Medium ladle (89g )  Large ladle (157g) | \|  \|  \|  \| \| --- \| --- \| --- \| |
| 30. Aterkik wet | Never or < 1 x per month  1 x per month  2- 3 x per month  1 x per week  XX  2-3 x per week  4-6 x per week  Every day | **Written in Number of ladles**  Small ladle (63 g)  Medium ladle (80g)  Large ladle (134g) | \|  \|  \|  \| \| --- \| --- \| --- \| |
| 31. Ful ( ye bakela) | Never or < 1 x per month  1 x per month  2- 3 x per month  1 x per week  XX  2-3 x per week  4-6 x per week  Every day | **Large**: Portion of g (full plate)  **Medium** : Portion of g (half plate) | \|  \|  \|  \| \| --- \| --- \| --- \| |

**Roots and tubers**

| Food item | Average consumption over the last month | Average consumption | Usual portion  size (gram) |
| --- | --- | --- | --- |
| 32. Kocho | Never or < 1 x per month  1 x per month  2- 3 x per month  1 x per week  XX  2-3 x per week  4-6 x per week  Every day | **Number of large slices ( g)**  Number of medium slices  (= 169 g )  Number of small slices ( g) | \|  \|  \|  \| \| --- \| --- \| --- \| |
| 33. Beet root stew | Never or < 1 x per mm  1 x per month  2- 3 x per month  1 x per week  XX  2-3 x per week  4-6 x per week  Every day | **Written in Number of ladles**  Small ladle (55 g)  Large spoon (49 g | \|  \|  \|  \| \| --- \| --- \| --- \| |
| 34. Sweet potato | Never or < 1 x per month  1 x per month  2- 3 x per month  1 x per week  XX  2-3 x per week  4-6 x per week  Every day | Number of pieces (1= g) | \|  \|  \|  \| \| --- \| --- \| --- \| |
| 35. Carrot with potato stew/Carrot be dinich wot | Never or < 1 x per month  1 x per month  2- 3 x per month  1 x per week  XX  2-3 x per week  4-6 x per week  Every day | **Written in Number of ladles**  Medium ladle (126 g)  Small ladle (91g)  Large spoon ( 67g) | \|  \|  \|  \| \| --- \| --- \| --- \| |

**Vegetables**

| Food item | Average consumption over the last month | Average consumption | Usual portion  size (gram) |
| --- | --- | --- | --- |
| 36. Cabbage (tikilgomen) | Never or < 1 x per month  1 x per month  2- 3 x per month  1 x per week  XX  2-3 x per week  4-6 x per week  Every day | **Written in Number of ladles**  Small ladle (90g)  Medium ladle (135 g  Large spoon (47 g) | \|  \|  \|  \| \| --- \| --- \| --- \| |
| 37. Kale (tikurgomen) | Never or < 1 x per month  1 x per month  2- 3 x per month  1 x per week  XX  2-3 x per week  4-6 x per week  Every day | **Written in Number of ladles**  Small ladle (53 g)  Medium ladle (103 g  Large ladle (151 g) | \|  \|  \|  \| \| --- \| --- \| --- \| |
| 38. Kosta | Never or < 1 x per month  1 x per month  2- 3 x per month  1 x per week  XX  2-3 x per week  4-6 x per week  Every day | **Written in Number of ladles**  Small ladle (___g)  Large spoon (___g) | \|  \|  \|  \| \| --- \| --- \| --- \| |
| 39. Tomato sauce/Timatimsilsih | Never or < 1 x per month  1 x per month  2- 3 x per month  1 x per week  XX  2-3 x per week  4-6 x per week  Every day | **Written in Number of ladles**  Small ladle (94 g)  Medium ladle (112 g  Large spoon (70 g) | \|  \|  \|  \| \| --- \| --- \| --- \| |
| 40. Tomato chopped/Timatim kurtih | Never or < 1 x per month  1 x per month  2- 3 x per month  1 x per week  XX  2-3 x per week  4-6 x per week  Every day | **Written in Number of ladles**  Small ladle (56 g)  Medium ladle (70g  Large spoon (37 g) | \|  \|  \|  \| \| --- \| --- \| --- \| |

| 41. Pumpkin stew/duba wot | Never or < 1 x per month  1 x per month  2- 3 x per month  1 x per week  XX  2-3 x per week  4-6 x per week  Every day | **Written in Number of ladles**  Small ladle (102 g)  Medium ladle (201 g  Large ladle (307 g) | \|  \|  \|  \| \| --- \| --- \| --- \| |
| --- | --- | --- | --- | --- | --- | --- |
| 42. Fosoliya | Never or < 1 x per month  1 x per month  2- 3 x per month  1 x per week  XX  2-3 x per week  4-6 x per week  Every day | **Written in Number of ladles**  Small ladle (66 g)  Large spoon (47g )  Soup spoon (33 g) | \|  \|  \|  \| \| --- \| --- \| --- \| |
| 43. Vegetable soup / ye atikiltihshorba | Never or < 1 x per month  1 x per month  2- 3 x per month  1 x per week  XX  2-3 x per week  4-6 x per week  Every day | **Written in Number of ladles**  Small ladle (___g)  Medium ladle (___g  Large ladle (___g) | \|  \|  \|  \| \| --- \| --- \| --- \| |

**Fruits**

| Food item | Average consumption over the last month | Average consumption | Usual portion  size (gram) |
| --- | --- | --- | --- |
| 44. Banana | Never or < 1 x per month  1 x per month  2- 3 x per month  1 x per week  XX  2-3 x per week  4-6 x per week  Every day | Number of pieces (1=94 g) | \|  \|  \|  \| \| --- \| --- \| --- \| |
| 45. Orange | Never or < 1 x per month  1 x per month  2- 3 x per month  1 x per week  XX  2-3 x per week  4-6 x per week  Every day | Number of pieces (1= 146 g) | \|  \|  \|  \| \| --- \| --- \| --- \| |
| 46. Mango | Never or < 1 x per month  1 x per month  2- 3 x per month  1 x per week  XX  2-3 x per week  4-6 x per week  Every day | Number of pieces (1= 212 g) | \|  \|  \|  \| \| --- \| --- \| --- \| |
| 47. Avocado | Never or < 1 x per month  1 x per month  2- 3 x per month  1 x per week  XX  2-3 x per week  4-6 x per week  Every day | Number of pieces (1= 185 g)  Small sized ladle ( g)  Spoon (large sized ) (= g) | \|  \|  \|  \| \| --- \| --- \| --- \| |
| 48. Papaya | Never or < 1 x per month  1 x per month  2- 3 x per month  1 x per week  XX  2-3 x per week  4-6 x per week  Every day | Large portion (=475 g)   \|  \|  \| \| --- \| --- \|   Medium portion (=237g)   \|  \|  \| \| --- \| --- \|   Small portion (=129 g)   \|  \|  \| \| --- \| --- \| |  |

**Egg**

| Food item | Average consumption over the last month | Average consumption | Usual portion  size (gram) |
| --- | --- | --- | --- |
| 49. Chicken eggs | Never or < 1 x per month  1 x per month  2- 3 x per month  1 x per week  XX  2-3 x per week  4-6 x per week  Every day | Number of pieces (1= 50 g) | \|  \|  \|  \| \| --- \| --- \| --- \| |

**Milk and dairy**

| Food item | Average consumption over the last month | Average consumption | Usual portion  size (gram) |
| --- | --- | --- | --- |
| 50. Milk: cow’s | Never or < 1 x per month  1 x per month  2- 3 x per month  1 x per week  XX  2-3 x per week  4-6 x per week  Every day | **Show pictures**  Number of large glasses (350 ml  Number of medium glasses (275 ml  Number of medium cups ( ml  Number of small cups(131 ml) | \|  \|  \|  \| \| --- \| --- \| --- \| |
| 51. Cheese | Never or < 1 x per month  1 x per month  2- 3 x per month  1 x per week  XX  2-3 x per week  4-6 x per week  Every day | large spoon (32 g)  Small ladle (59 g)  Medium ladle (106 g) |  |

| 52. Yoghurt | Never or < 1 x per month  1 x per month  2- 3 x per month  1 x per week  XX  2-3 x per week  4-6 x per week  Every day | **Show pictures**  Number of large yoghurt glasses ( 475ml)  Number of medium yoghurt glasses (350 ml  Number of small yoghurt glasses (295 ml | \|  \|  \|  \| \| --- \| --- \| --- \| |
| --- | --- | --- | --- | --- | --- | --- |

**Fish and products based on fish**

| Food item | Average consumption over the last month | Average consumption | Usual portion  size (gram) |
| --- | --- | --- | --- |
| **Fish**  53. Fried fish (asatibs)  54. Asa stew (Asa wot)  55. Asa stew (Asa wot)  56. Asa gulash  57. Tuna  58. Asa lebleb  59. Asa kotelet | Never or < 1 x per month  1 x per month  2- 3 x per month  1 x per week  XX  2-3 x per week  4-6 x per week  Every day | Piece fish of g (=100 g : fillet)  (feleto)  Fried fish = 406 g  Tuna = 75 g | \|  \|  \|  \| \| --- \| --- \| --- \| |

**Meat and poultry**

| Food item | Average consumption over the last month | Average consumption | Usual portion size (gram) |
| --- | --- | --- | --- |
| **Meat**  60. Sigawotih | Never or < 1 x per month  1 x per month  2- 3 x per month  1 x per week  XX  2-3 x per week  4-6 x per week  Every day | **Written in Number of ladles**  Small ladle (54 g)  Medium ladle (103 g  Large ladle (158 g) | \|  \|  \|  \| \| --- \| --- \| --- \| |
| 61. Kitfo | Never or < 1 x per month  1 x per month  2- 3 x per month  1 x per week  XX  2-3 x per week  4-6 x per week  Every day | **Written in Number of ladles**  Small ladle (60 g)  Medium ladle (80 g  Large ladle (118 g) | \|  \|  \|  \| \| --- \| --- \| --- \| |
| 62. Gored gored | Never or < 1 x per month  1 x per month  2- 3 x per month  1 x per week  XX  2-3 x per week  4-6 x per week  Every day | **Written in Number of ladles**  Small ladle ( g)  Medium ladle ( g  Large ladle ( g) | \|  \|  \|  \| \| --- \| --- \| --- \| |
| 63. Ye berehtibsih | Never or < 1 x per month  1 x per month  2- 3 x per month  1 x per week  XX  2-3 x per week  4-6 x per week  Every day | **Written in Number of ladles**  Small ladle (32 g)  Medium ladle (51 g  Large ladle (78 g) | \|  \|  \|  \| \| --- \| --- \| --- \| |
| 64. Minchet | Never or < 1 x per month  1 x per month  2- 3 x per month  1 x per week  XX  2-3 x per week  4-6 x per week  Every day | **Written in Number of ladles**  Small ladle ( g)  Medium ladle ( g  Large ladle ( g) | \|  \|  \|  \| \| --- \| --- \| --- \| |
| 65. Kikil | Never or < 1 x per month  1 x per month  2- 3 x per month  1 x per week  XX  2-3 x per week  4-6 x per week  Every day | **Written in Number of ladles**  Small ladle ( g)  Medium ladle ( g  Large spoon ( g) | \|  \|  \|  \| \| --- \| --- \| --- \| |
| 66. Ye begih tibs | Never or < 1 x per month  1 x per month  2- 3 x per month  1 x per week  XX  2-3 x per week  4-6 x per week  Every day | **Written in Number of ladles**  Small ladle (32 g)  Medium ladle (51 g  Large spoon (78 g) | \|  \|  \|  \| \| --- \| --- \| --- \| |
| **Poultary (ye dorosiga)**  67. Chicken stew/doro wet  68. Doro alicha  69. Doro tibs wot | Never or < 1 x per month  1 x per month  2- 3 x per month  1 x per week  XX  2-3 x per week  4-6 x per week  Every day | **Written in Number of ladles**  Small ladle ( g)  Medium ladle ( g  Large spoon ( g) |  |

**Fat and oil**

| Food item | Average consumption over the last month | Average consumption | Usual portion size (gram) |
| --- | --- | --- | --- |
| **70. Butter** | Never or < 1 x per month  1 x per month  2- 3 x per month  1 x per week  XX  2-3 x per week  4-6 x per week  Every day | **Written in Number of ladles**  Small spoon ( g)  Medium spoon ( g  Large spoon ( g) | \|  \|  \|  \| \| --- \| --- \| --- \| |
| 71. Oil (type of oil)  Plant/palm/ saturated/ yerega | Never or < 1 x per month  1 x per month  2- 3 x per month  1 x per week  XX  2-3 x per week  4-6 x per week  Every day | Small spoon ( g)  Medium spoon ( g  Large spoon ( g) | \|  \|  \|  \| \| --- \| --- \| --- \| |

**Sweets**

| Food item | Average consumption over the last month | Average consumption | Usual portion size (gram) |
| --- | --- | --- | --- |
| 72. Honey  73. Jam | Never or < 1 x per month  1 x per month  2- 3 x per month  1 x per week  XX  2-3 x per week  4-6 x per week  Every day | **Written in Number of ladles**  Small spoon (8 g)  Medium spoon (12 g  Large spoon (23 g) | \|  \|  \|  \| \| --- \| --- \| --- \| |
| 74. Sugar | Never or < 1 x per month  1 x per month  2- 3 x per month  1 x per week  XX  2-3 x per week  4-6 x per week  Every day | **Written in Number of ladles**  Small spoon ( 4g)  Medium spoon ( 7g  Large spoon (16 g) | \|  \|  \|  \| \| --- \| --- \| --- \| |

**Drinks**

| **Non-Alcoholic drinks**  Food item | Average consumption over the last month | Average consumption | Usual portion size (gram) |
| --- | --- | --- | --- |
| 75. Coffee | Never or < 1 x per month  1 x per month  2- 3 x per month  1 x per week  XX  2-3 x per week  4-6 x per week  Every day | Number of coffee cups/ show images  Small cup (___g)  Medium cup (___g)  Large cup (___g) | \|  \|  \|  \| \| --- \| --- \| --- \| |
| 76. Tea | Never or < 1 x per month  1 x per month  2- 3 x per month  1 x per week  XX  2-3 x per week  4-6 x per week  Every day | Number of tea cups/ show images  Small cup(___g)  Medium cup (___g)  Large cup (___g) | \|  \|  \|  \| \| --- \| --- \| --- \| |
| 77. Mekiyato/ buna be wetet | Never or < 1 x per month  1 x per month  2- 3 x per month  1 x per week  XX  2-3 x per week  4-6 x per week  Every day | Number of cups/ show images  Small cup(___g)  Medium cup (___g)  Large cup (___g) | \|  \|  \|  \| \| --- \| --- \| --- \| |

| **Soft drinks**  78. Mirinda  79. Fanta  80. Pepsi  81. Coca-cola | Never or < 1 x per month  1 x per month  2- 3 x per month  1 x per week  XX  2-3 x per week  4-6 x per week  Every day | Number of glasses/  Small glasses (400ml)  Medium glasses (275ml)  Large glasses (250 ml) | \|  \|  \|  \| \| --- \| --- \| --- \| |
| --- | --- | --- | --- | --- | --- | --- |
| **Alcoholic drinks**  82. Beer | Never or < 1 x per month  1 x per month  2- 3 x per month  1 x per week  XX  2-3 x per week  4-6 x per week  Every day | Number of cans or bottles of 330ml  Number of cans or bottles of (500ml) | \|  \|  \|  \| \| --- \| --- \| --- \| |
| 83. Tella  84. Tej  85. Arake  86. borde | Never or < 1 x per month  1 x per month  2- 3 x per month  1 x per week  XX  2-3 x per week  4-6 x per week  Every day | Number of glasses/  Small glasses (400ml)  Med glasses (275ml)  Large glasses (250 ml) | \|  \|  \|  \| \| --- \| --- \| --- \| |

**Fast foods and pastry**

| Food item | Average consumption over the last month | Average consumption | Usual portion size (gram) |
| --- | --- | --- | --- |
| 87. Burger | Never or < 1 x per month  1 x per month  2- 3 x per month  1 x per week  XX  2-3 x per week  4-6 x per week  Every day | Number of 1 (1)pieces = 376 g  Number of ½ or half pieces | \|  \|  \|  \| \| --- \| --- \| --- \| |
| 88. Pizza | Never or < 1 x per month  1 x per month  2- 3 x per month  1 x per week  XX  2-3 x per week  4-6 x per week  Every day | Small slice (1/4 full : 110g)  Medium slice (1/2 full :184g )  Full/ one pizza = 308 g | \|  \|  \|  \| \| --- \| --- \| --- \| |
| 89. Cake ( types of cake) | Never or < 1 x per month  1 x per month  2- 3 x per month  1 x per week  XX  2-3 x per week  4-6 x per week  Every day | Number of large slice = g | \|  \|  \|  \| \| --- \| --- \| --- \| |
